# Retraction Note: Association between cytochrome P4501A1 (*CYP1A1*) gene polymorphisms and the risk of renal cell carcinoma: a meta-analysis

**DOI:** 10.1038/s41598-025-86862-7

**Published:** 2025-01-22

**Authors:** Fan-dong Meng, Ping Ma, Cheng-guang Sui, Xin Tian, You-hong Jiang

**Affiliations:** MolecularOncology Department of Cancer Research Institution, The First Hospital of China Medical University, Shenyang, 110001 China

Retraction of: *Scientific Reports* 10.1038/srep08108, published online 29 January 2015

The Editors have retracted this Article.

Following publication, the misnaming of the “Begg’s” funnel plot as “Beggar’s” in the Article was brought to the attention of the Editors. Investigation by the Editors have uncovered further issues, including significant textual overlaps with pre-existing work, such as^1,2^. Authors have not responded to the Editors’ requests for clarification and the Editors no longer have confidence in the claims of the Article.

Fan-dong Meng, Xin Tian and You-hong Jiang did not respond to correspondence from the Editors about this retraction. The Editors have been unable to establish the current email addresses for Ping Ma and Cheng-guang Sui.
